# Dietary lipid content reorganizes gut microbiota and probiotic *L*. *rhamnosus* attenuates obesity and enhances catabolic hormonal milieu in zebrafish

**DOI:** 10.1038/s41598-017-05147-w

**Published:** 2017-07-17

**Authors:** Silvia Falcinelli, Ana Rodiles, Azadeh Hatef, Simona Picchietti, Lina Cossignani, Daniel L. Merrifield, Suraj Unniappan, Oliana Carnevali

**Affiliations:** 1 Dipartimento di Scienze della Vita e dell’Ambiente, Università Politecnica delle Marche, Ancona, Italy; 2 Aquatic Animal Nutrition and Health Research Group, School of Biological and Marine Sciences, Plymouth University, Plymouth, PL4 8AA United Kingdom; 3 Department of Veterinary Biomedical Sciences, Laboratory of Integrative Neuroendocrinology, Western College of Veterinary Medicine, University of Saskatchewan, 52 Campus Drive, Saskatoon, Saskatchewan S7N 5B4 Canada; 4 Department for Innovation in Biological, Agro-food and Forest Systems (DIBAF), University of Tuscia, Viterbo, Italy; 5 Dipartimento di Scienze Farmaceutiche, Sezione di Scienze degli Alimenti e Nutrizione, Università degli Studi di Perugia, Perugia, Italy

## Abstract

In the present study, we explored whether dietary lipid content influences the gut microbiome in adult zebrafish. Diets containing three different lipid levels (high [HFD], medium [MFD], and low [LFD]) were administered with or without the supplementation of *Lactobacillus rhamnosus* (P) to zebrafish in order to explore how the dietary lipid content may influence the gut microbiome. Dietary lipid content shifted the gut microbiome structure. The addition of *L*. *rhamnosus* in the diets, induced transcriptional reduction of orexigenic genes, upregulation of anorexigenic genes, and transcriptional decrease of genes involved in cholesterol and triglyceride (TAG) metabolism, concomitantly with lower content of cholesterol and TAG. Probiotic feeding also decreased nesfatin-1 peptide in HFD-P and attenuated weight gain in HFD-P and MFD-P fed zebrafish, but not in LFD-P group. Intestinal ultrastructure was not affected by dietary fat level or probiotic inclusion. In conclusion, these findings underline the role of fat content in the diet in altering gut microbiota community by shifting phylotype composition and highlight the potential of probiotics to attenuate high-fat diet-related metabolic disorder.

## Introduction

In recent decades, the incidences of metabolic syndrome (MS) have dramatically increased globally^1^. MS involves a complex cluster of linked factors which increases the risk of cardiovascular diseases, type 2 diabetes and obesity^1^. Such diseases are thought to be induced, at least in part, by high-fat-diets (HFD) which are increasingly associated with modern global eating habits^2, 3^. Accumulating evidence suggests that HFD negatively modulate the composition of the gut microbiota, a condition known as dysbiosis^4, 5^. Recently, the gut microbiota has emerged as a key factor which regulates host metabolism and different gut microbiome phenotypes are associated with chronic diseases such as inflammatory bowel diseases and the development of MS symptoms^4, 6, 7^.

Over the last decade probiotics have gained popularity with a growing body of research demonstrating that, through the modulation of the microbiota, they are able to prevent and treat different gastrointestinal diseases and to modify host nutrient metabolism and energy homeostasis^8, 9^. Recent studies have focused on the possibility to therapeutically target the gut microbiota by administering food additives in an attempt to reduce MS symptoms^1, 2^. Consequently, there is a pressing need to understand the molecular mechanisms by which the host responds to probiotics when fed a HFD, since we recently reported that probiotics may play a therapeutic role in the treatment of lipid disorders induced by elevated fat^10^.

The present study was therefore undertaken to evaluate how dietary lipid content may influence the gut microbiota and metabolism in adult zebrafish, and if a probiotic may alleviate potential dysbiosis and secondary host metabolic disruptions. The probiotic *Lactobacillus rhamnosus*, which we have demonstrated affects appetite control and lipid metabolism in zebrafish^9, 10^ was used as a probiotic supplement for adult zebrafish fed either a high fat diet (HFD; 15% lipid), medium fat diet (MFD; 10% lipid), or a low fat diet (LFD; 5% lipid). In order to accomplish this, through an integrated approach, we evaluated the changes in gut microbiota community and host gene expression of zebrafish exposed to diets with different lipid contents with and without the supplementation of *L*. *rhamnosus*. The expression of a wide network of genes involved in lipid metabolism and appetite control, together with the synthesis of orexigenic and anorexigenic peptides in the zebrafish gut was examined. Moreover, we evaluated the effect of *L*. *rhamnosus*, and different dietary lipid contents, on intestinal epithelial architecture, total body cholesterol and TAG content and total body weight.

## Results

### Diet fat content and probiotic feeding modulates the expression of genes involved in appetite control

**Figure 1:**
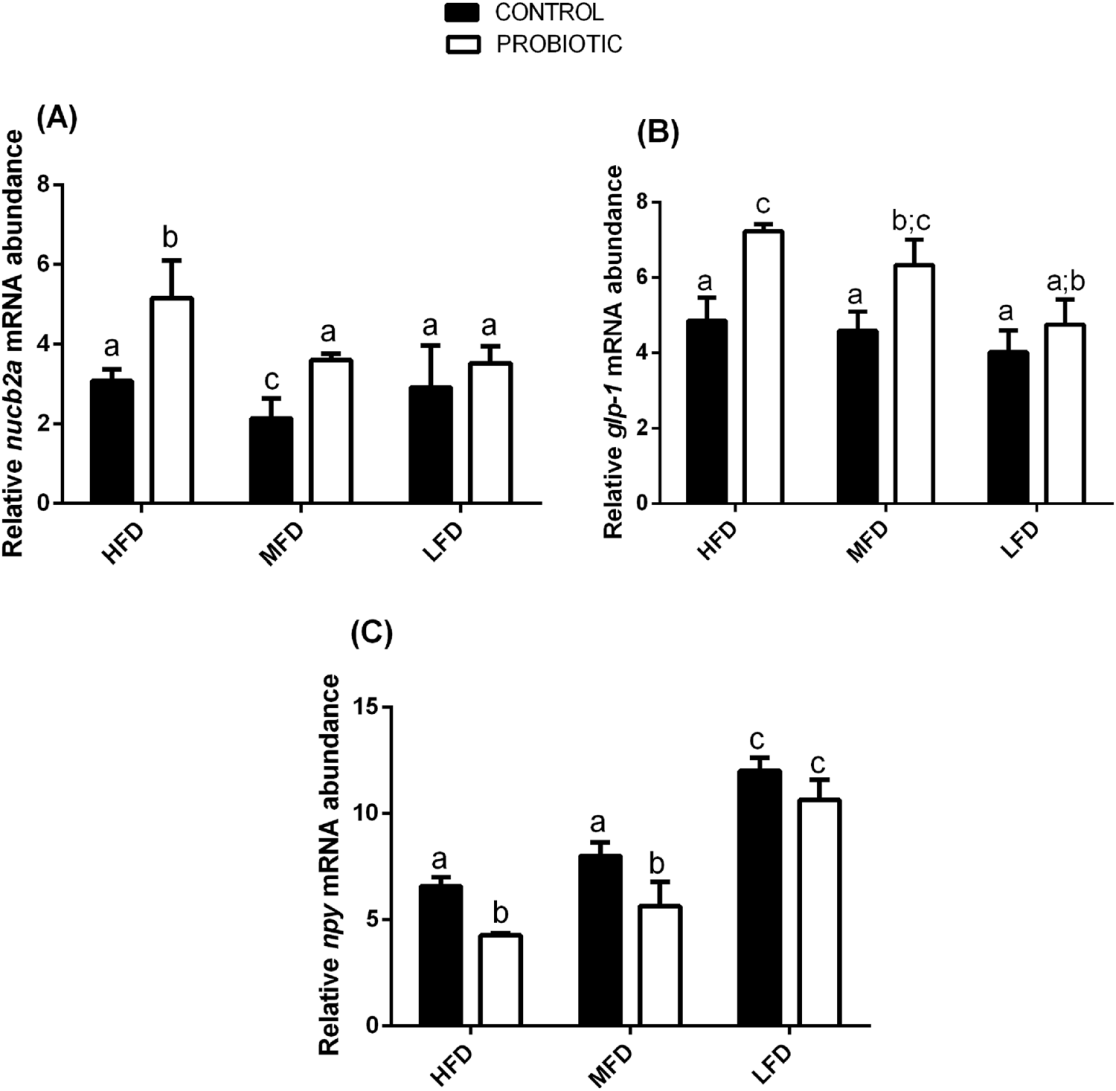
*L*. *rhamnosus* modulates the expression of genes involved in the regulation of appetite. Relative *nucb2a* (**A**), *gcga* (**B**), *npy* (**C**), gene expression normalized against *β-act* and *rplp*, in fish fed a HFD-C, MFD-C and LFD-C and fish fed a HFD-P, MFD-P and LFD-P. Assays were performed in triplicate. Values with different superscript letters are significantly different (*p* < 0.05).

In order to investigate the effects of probiotic administration in zebrafish fed a HFD (15% dietary lipid), MFD (10% dietary lipid) and LFD (5% dietary lipid), we evaluated the expression of genes associated with the appetite, by performing Real Time PCR on 9 samples, each sample made of a pool of 8 adult. NUCB2/nesfatin-1 enhances insulin release from β-cells and reduces food intake^11–14^. Our analysis revealed that with medium content of fat in the diet (MFD-C), significant lower *nucb2a* gene expression was observed compared to high (HFD-C) and low (LFD-C) content of fat in the diets (Fig. 1A) (*p* < 0.05). The addition of the probiotic to the diets significantly increased *nucb2a* gene expression in fish fed a HFD-P and MFD-P compared to HFD-C and MFD-C fed fish (*p* < 0.05), while no significant differences in mRNA levels of this gene was observed between LFD-C fed fish and LFD-P fed fish (Fig. 1A) (*p* > 0.05). Glucagon-like peptide 1, encoded by *gcga* and *gcgb* in zebrafish, has crucial role in stimulating insulin release and inhibiting appetite and is successfully used as an anti-diabetic drug^15^. Our results indicate that different dietary fat content did not affect the expression of this gene among HFD-C, MFD-C and LFD-C fed fish (Fig. 1B) (*p* > 0.05). However, the administration of the probiotic significantly up-regulated *gcga* gene expression in fish fed HFD-P and MFD-P compared to HFD-C and MFD-C fed fish (Fig. 1B) (*p* < 0.05). No significant differences in mRNA levels were observed between LFD-P and LFD-C fed fish (Fig. 1B) (*p* > 0.05). Neuropetide Y (NPY) stimulates food intake^16^. Our results show that the expression of the *npy* gene was modulated in correlation to fat content. *npy* mRNA level gradually and significantly decreased with increasing fat in the diet, with the lowest mRNA level in the fish fed HFD-C (Fig. 1C) (*p* < 0.05). In the probiotic treated groups, data showed a significant down-regulation of *npy* gene transcripts in HFD-P and MFD-P fed fish compared to HFD-C and MFD-C fed fish (*p* < 0.05), while no significant differences in *npy* mRNA was observed in LFD-C and LFD -P fed fish (Fig. 1C) (*p* > 0.05).

**Figure 2:**
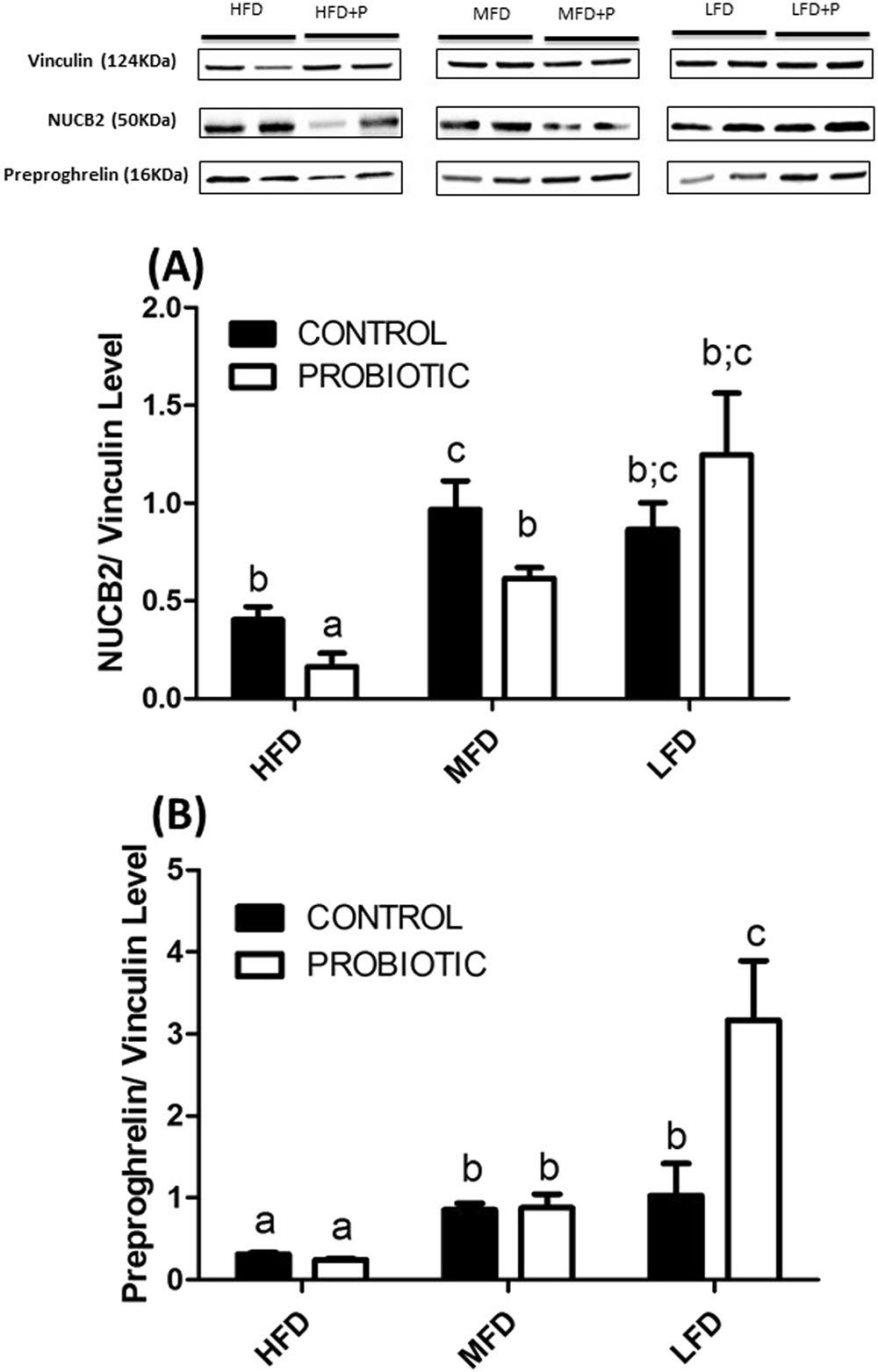
*L*. *rhamnosus* affects the protein expression of NUCB2 and preproghrelin in zebrafish trunk fed a HFD-P, MFD-P and LFD-P. A Western blot using the rabbit anti-nesfatin-1 antibody showed the presence of 50 kDa NUCB2 proteins in zebrafish trunk. NUCB2/Nesfatin-1 was significantly decreased in MFD-P and HFD-P fed fish compare to MFD-C and HFD-C (**A**). Graph (**B**) showed no differences between preproghrelin content in zebrafish fed a MFD-P and HFD-P compared to MFD-C and HFD-C fed fish. Preproghrelin protein was significantly increased in LFD-P compare to LFD-C. Values with different superscript letters are significantly different (*p* < 0.05).

Western blot (WB) analysis, perfomed on 4 samples, each sample made of a zebrafish trunk, using a rabbit anti-nesfatin-1 antibody demonstrated the presence of 50 kDa NUCB2, the precursor of nesfatin-1, in zebrafish trunk. NUCB2 peptide was significantly lower in HFD-C compared to diets with lower fat content (MFD-C and LFD-C) (Fig. 2A) (*p* < 0.05). With the addition of probiotic, the NUCB2 displayed a similar trend observed in the control groups, as well as it was significantly decreased in HFD-P and MFD-P fed fish compared to control diet. This reduction in NUCB2 is likely due to the secretion of the precursor peptide (NUCB2), and/or processed nesfatin-1 into circulation. The presence of a signal peptide enables the secretion of NUCB2 into circulation^17^. A band representing the processed nesfatin-1 was not visible in samples tested from any of the groups. This might be due to the lower, undetectable levels of nesfatin-1 in the trunk of zebrafish. To the best of our knowledge, only one study so far reported a processed nesfatin-1 band in a Western gel^18^, and that was feasible due to the use of protein from enriched cells/tissues that express nesfatin-1 in abundance. Further studies to determine gut specific relative abundance of NUCB2 versus nesfatin-1 in zebrafish are warranted. There were no differences in NUCB2 levels in LFD-P and LFD-C fed fish (Fig. 2A) (*p* > 0.05). In addition, Western blot analysis of zebrafish trunk with mouse anti-ghrelin antibody showed full-length preproghrelin protein (16 kDa). Preproghrelin levels significantly decreased in HFD-C fed fish compared to fish fed MFD-C and LFD-C (Fig. 2B) (*p* < 0.05). Preproghrelin protein was significantly increased in the LFD-P group compared to the LFD-C fed group, and this likely suggests less conversion of the precursor to processed orexigen ghrelin. There were no differences between preproghrelin content in zebrafish fed a MFD-P and HFD-P compared to fish fed a MFD-C and HFD-C (Fig. 2B) (*p* > 0.05). While inter individual variations in band intensities were observed, the normalized results with v inculin clearly indicate that precursors of nesfatin-1 increase while the fat content increase in the diet and ghrelin decreased when the fat content increase in the diet.

**Figure 3:**
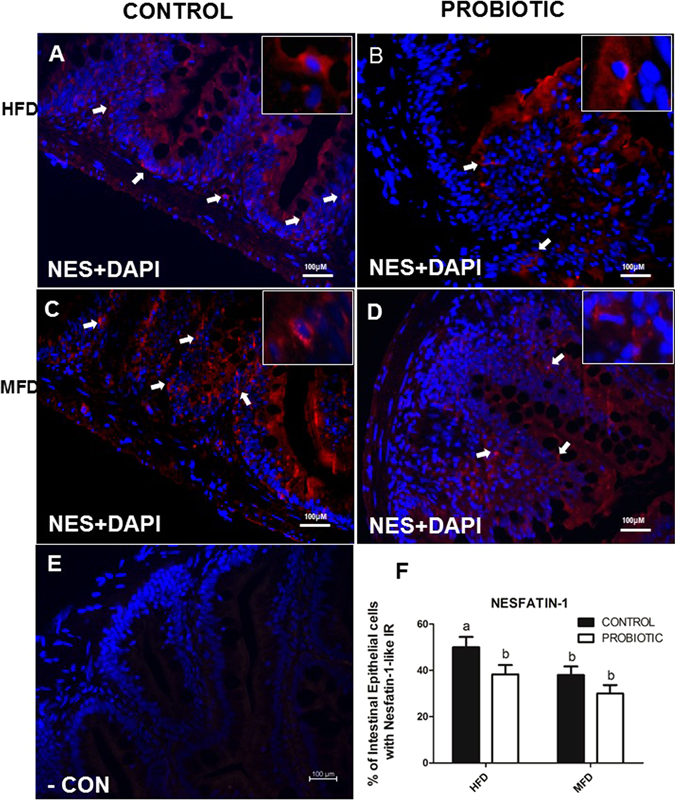
NUCB2/nesfatin-1-like ir in the gut of zebrafish fed with HDF-P and HFD-C compare to MFD-C. Cross sections of the zebrafish for nesfatin-1-like show higher abundance of nesfatin-1-like cell in HFD-C compared to HFD-P (3 F). For details on methods employed for percentage of positive intestinal epithelial cells calculation, please consult the Materials and Methods section. Values with different superscript letters are significantly different (*p* < 0.05).

**Figure 4:**
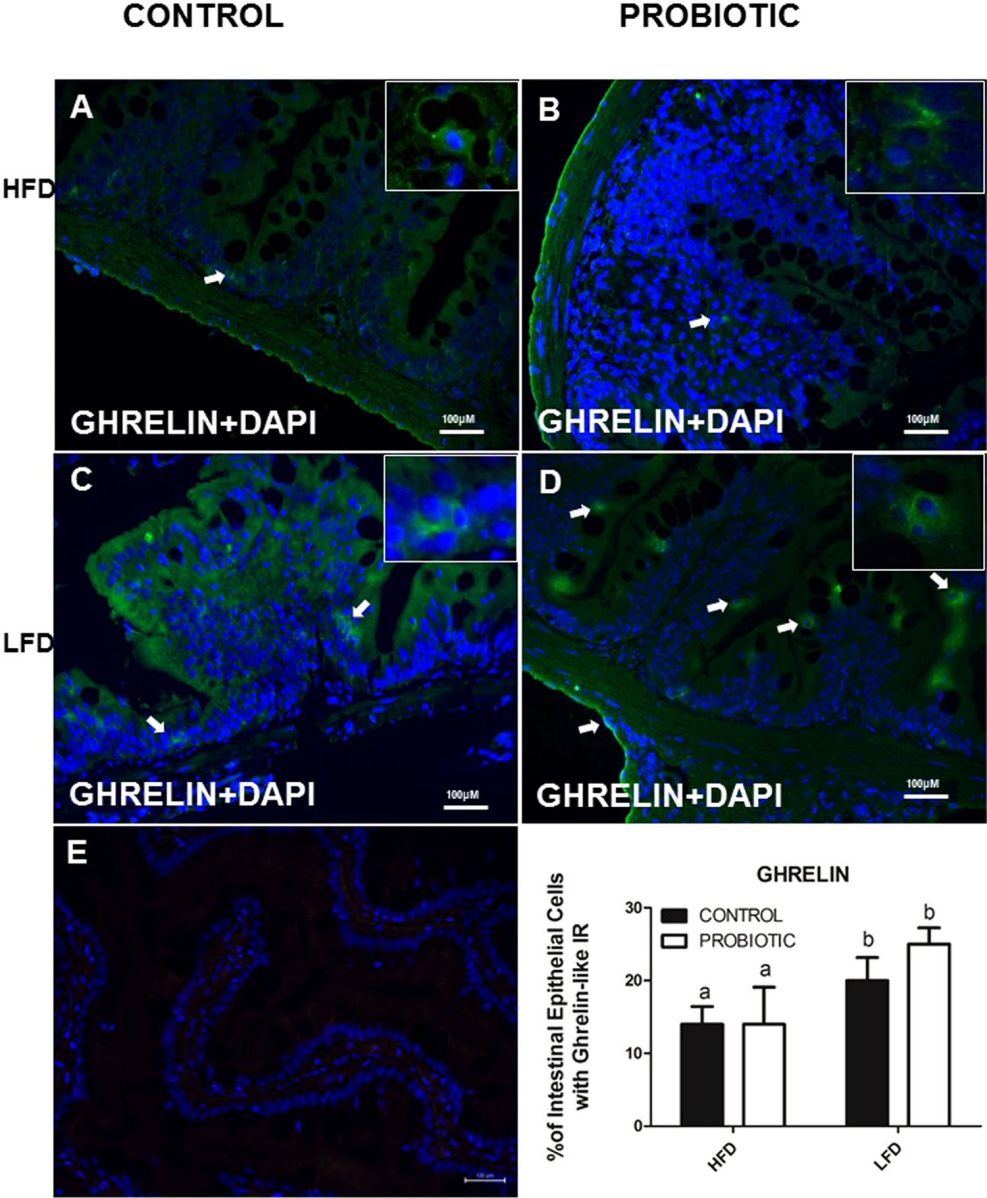
Ghrelin-like ir in the gut of zebrafish fed with HDF-P and HFD-C compare to LFD-C. Ghrelin positive cells did not show changes in the intestine of fish fed a LFD-C (4C) compare to LFD-P (4D) and HFD-C (4A) compare to HFD-P (4B). For details on methods employed for percentage of positive intestinal epithelial cells calculation, please consult the Materials and Methods section. Values with different superscript letters are significantly different (*p* < 0.05).

By analysing 4 zebrafish intestines (10 sections per fish), results showed that cross sections of 4 zebrafish intestine stained positive for nesfatin-1-like within rounded or flask-shaped cells (Fig. 3). Most of these cells were dispersed along the apical regions of the villi. Lightly stained processes emanating from the nesfatin-1 immunopositive cells are visible and appear to project towards the lumen (Fig. 3). Ghrelin-like immunoreactivity (ir) was detected in some cells in the villi (Fig. 4). DAPI stained the nuclei of nesfatin-1 and ghrelin- like ir cells and non-ir cells (Figs. 3 and 4). No immunoreactivity was observed in sections treated with the secondary antibody alone (Figs. 3E and 4E). Quantitative analysis of immunopositive cells to nesfatin-1 show that the number of positive cells to nesfatin-1 is higher in HFD compare to MFD in control group. Similar to our result in WB, immunoreactive cells to nesfatin-1 was higher in control compared to probiotic group in HFD (Fig. 3E). The abundance of Ghrelin positive cells were higher in the LFD group compared to the HFD group (Fig. 4F). However, there were no significant differences between HFD-P and HFD-C groups (Fig. 4F) (*p* > 0.05).

### Diet fat content and probiotic modulate the expression of genes involved in cholesterol metabolism and decrease total body cholesterol in zebrafish fed HFD and MFD, but not in LFD fed fish

To test the hypolipidemic activity of *L*. *rhamnosus* in adult zebrafish fed with HFD, MFD and LFD a wide network of genes which regulate cholesterol metabolism were evaluated from 9 samples per group, each sample made of a pool of 8 adult. Both fish and mammals use triglycerides and cholesterol derived from the diet as a main source of energy which can be released quickly on demand^16^. However, high serum cholesterol levels cause several metabolic disorders including type 2 diabetes, atherosclerosis and hypertension^19^. Hepatocyte nuclear factor 4 alpha (HNF4a) controls a wide network of genes and is able to enhance genes whose products are implicated in cholesterol, fatty acid and amino acid metabolism^20^, while Niemann-Pick C1-Like 1 (NPC1L) is a transmembrane protein localized at the apical membrane of enterocytes involved in sterol transport^20, 21^.

**Figure 5:**
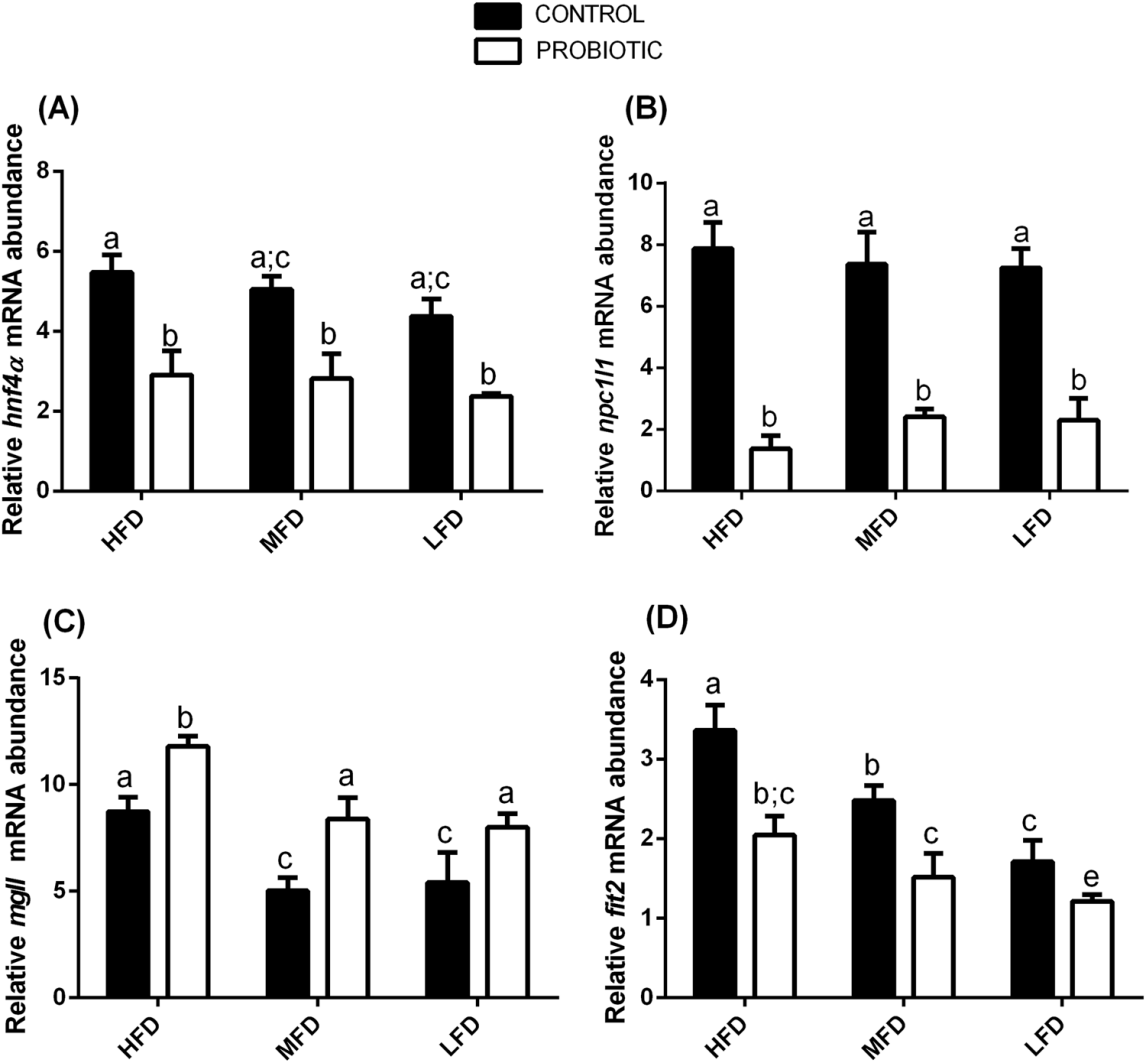
*L*. *rhamnosus* modulates the expression of genes involved in lipid metabolism. Relative *hnf4a* (**A**), *npc1l1* (**B**), *mgll* (**C**) and *fit2* (**D**), gene expression normalized against *β-act* and *rplp*, in fish fed a HFD-C, MFD-C and LFD-C and fish fed a HFD-P, MFD-P and LFD-P. Assays were performed in triplicate. Values with different superscript letters are significantly different (*p* < 0.05).

Our *hnf4a* and *npc1l1* gene expression analysis showed that the transcript level did not vary in relation to dietary fat content; no significant differences were observed among HFD-C, MFD-C, LFD-C fed fish (Fig. 5A,B) (*p* > 0.05). However, the addition of probiotic *L*. *rhamnosus* significantly decreased the gene expression of *hnf4a* in HFD-P, MFD-P and LFD-P-fed fish compared to the respective controls (Fig. 5A) (*p* < 0.05). Remarkably, *npc1l1* transcript levels decreased by almost 4-fold in HFD-P, MFD-P, LFD-P fed fish compared to HFD-C, MFD-C, LFD-C fed fish (Fig. 5B) (*p* < 0.05).

Since we reported a down-regulation of expression of genes whose products are involved in the cholesterol metabolism, we wanted to further investigate the effect of dietary *L*. *rhamnosus* supplementation on total body cholesterol content to test its effects 9 zebrafish samples per group, each sample made of a pool of adult until reaching 300 mg of tissue.

High Performance Liquid Chromatography (HPLC) analyses performed on total body, evidenced that the cholesterol content does not change in correlation of the fat content in the diet: HFD-C fed fish (6.2 ± 0.4 µg mg^−1^), MFD-C (7.6 ± 5.5 µg mg^−1^), LFD-C (5.3 ± 0.4 µg mg^−1^) (*p* > 0.05). However, comparing the groups fed probiotic-enriched and probiotic-free diets, zebrafish fed a HFD*-*P had significantly lower content of total cholesterol (3.1 ± 0.2 µg mg^−1^) compared to HFD-C fed fish (6.2 ± 0.4 µg mg^−1^) (*p* < 0.05). In addition, results showed a significantly lower total cholesterol content in zebrafish fed a MFD-P (5.5 ± 0.3 µg mg^−1^) compared to the total cholesterol of the zebrafish fed a MFD-C (7.6 ± 5.5 µg mg^−1^) confirming results of our previous work conducted on zebrafish larvae^10^. However, no significant differences were found in total cholesterol content between zebrafish fed a LFD-P (4.9 ± 1.1 µg mg^−1^) and zebrafish fed a LFD-C (5.3 ± 0.4 µg mg^−1^) (*p* > 0.05).

### Dietary fat content and probiotic supplementation modulates the expression of genes involved in TAG metabolism and decreases the total body TAG in zebrafish fed a HFD and MFD, but not in LFD fed fish

Since we reported compelling evidence of the inflection of cholesterol metabolism, we wanted to further elucidate the probiotic’s role on TAG metabolism in fish fed with different fat levels (analysis were performed on 9 samples per group, each sample made of a pool of 8 adult). TAG needs to be present in the body since is the major source of lipid storage^22, 23^. However, a high TAG content can lead to an excess of fat storage^22^. Our results showed that the fat content led to a variation in *mgll* gene transcript levels among fish fed the different diets. In fact, *mgll*, a gene which encodes a serine hydrolase involved in the breakdown of TAG to free fatty acids and monoacylglycerols^24^, was significantly up-regulated in HFD-C fed fish compared to MFD-C and LFD-C fed fish (Fig. 5C) (*p* < 0.05). Interestingly, the comparison among groups fed probiotic-enriched and probiotic-free diets revealed that the supplementation of the probiotic to the diet significantly up-regulated *mgll* gene transcripts in HFD-P, MFD-P and LFD-P fed fish compared to HFD-C, MFD-C and LFD-C fed fish (Fig. 5C) (*p* < 0.05).

The analysis of *fit2* gene, which codifies for a transmembrane proteins (FIT2), which distribute TAG into lipid droplets^16, 25, 26^, showed that the transcription process of such mRNA is fat-dependent. In fact, *fit2* gene transcript gradually and significantly decrease concomitantly with the decreasing of fat content in the diet, highlighting the higher transcript level in fish fed a HFD-C (Fig. 5D) (*p* < 0.05). The comparison among group fed a diets probiotic enriched or not, showed a significantly down-regulation of *fit2* gene transcripts in HFD-P, MFD-P and LFD-P probiotic fed fish compared to HFD-C, MFD-C and LFD-C fed fish (Fig. 5D) (*p* < 0.05).

HPLC analysis performed on total body of 9 samples per group (each sample made of a pool of adult until reaching 300 mg of tissue) evidenced that TAG content significantly decrease with the decreasing of the fat in the diet; HFD-C (92.5 ± 0.8 µg mg^−1^), MFD-C (83.7 ± 3.8 µg mg^−1^) and LFD-C (67.6 ± 0.3 µg mg^−1^) (*p* < 0.05). Interestingly, comparing the group fed a diet probiotic enriched or not, results shows that zebrafish fed a HFD-P had significantly lower content of total TAG (73.5 ± 0.4 µg mg^−1^) compared to the fish fed a HFD-C (92.5 ± 0.8 µg mg^−1^). Similarly, significantly lower total TAG content in zebrafish fed a MFD-P (66.5 ± 4.9 µg mg^−1^) compared to fish fed a MFD-C (83.7 ± 3.8 µg mg^−1^) was observed; while no significant difference was observed in total TAG content between the LFD-C (67.6 ± 0.3 µg mg^−1^) and LFD-P (66.1 ± 0.3 µg mg^−1^) groups (*p* < 0.05).

### Dietary fat content and probiotic *L*. *rhamnosus* does not impact intestinal ultrastructure

**Figure 6:**
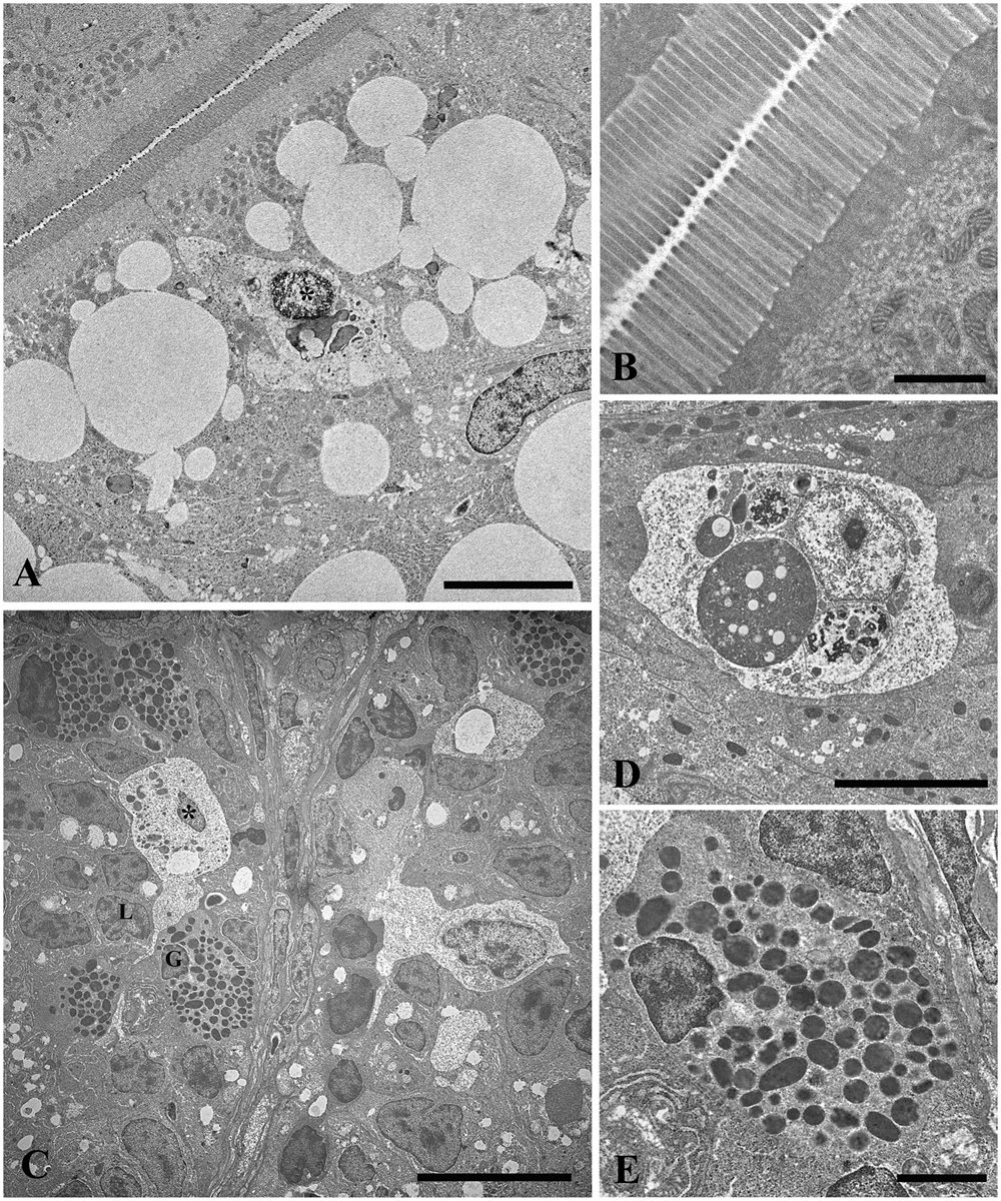
Elevated fat content increases lipid droplets, but did not alter intestinal mucosal epithelium in zebrafish fed a HFD, MFD and LFD. TEM micrograph showing large lipid droplets accumulation in the apical epithelium of enterocytes and intraepithelial macrophages in HFD-C fed fish (**A**). Intact brush border in HFD-C fed fish, (**B**). Macrophages in MFD-C fed fish in contact with the cell membrane of infiltrated lymphocytes and granulocytes (**C**). Intraepithelial macrophages with typical features of phagocytic cells in LFD-C fed fish (**D**). Magnification of intra-epithelium granulocytes in MFD-C fed fish (**E**). L = lymphocyte; G = granulocyte; Asterisk = macrophage. Scale bars: A = 5 µm, B = 1 µm, C = 10 µm, D = 5 µm, E = 2 µm.

The intestine of adult zebrafish is a folded duct that occupies the abdominal cavity and is composed of three segments involved in different processes^27^. In particular, as the anterior segment is marked by the presence of proteins involved in the absorption of lipids^27^, this intestinal region has selected for ultrastructural analysis. Ultrathin sections of the anterior intestine of zebrafish fed a HFD-C (Fig. 6A,B) revealed an intact brush border and the presence of large lipid droplet accumulations in the apical cytoplasm of the enterocytes. Some macrophages (Fig. 6A) with spherical or ovoidal shapes, euchromatic nuclei with marginal heterochromatin and fine-granular cytoplasm were infiltrated in the epithelium. Similarly, the MFD-C fed fish also contained macrophages with typical features of phagocytic cells (Fig. 6C), mainly localized in the basolateral epithelium and seldom in the apical zone. These cells often contacted the cell membranes of infiltrated lymphocytes and granulocytes (Fig. 6C). Some macrophages and granulocytes, the latter characterized by heterochromatic nuclei and cytoplasm containing numerous granules housing homogeneous and electron-dense material (Fig. 6D,E), were also infiltrated in the epithelium of LFD-C fed fish. The morphology of the intestinal epithelium was not affected by the probiotic. The intestine of zebrafish fed a HFD-P, MFD-P and LFD-P diets, similarly to their respective controls, had well organized microvilli and no cell debris in the lumen, an intact intestinal epithelium with intracellular tight junctions which preserve epithelial integrity (See supplemental information S.I. Fig. 1).

### Dietary fat content and probiotic *L*. *rhamnosus* affect microbiome gut community

**Figure 7:**
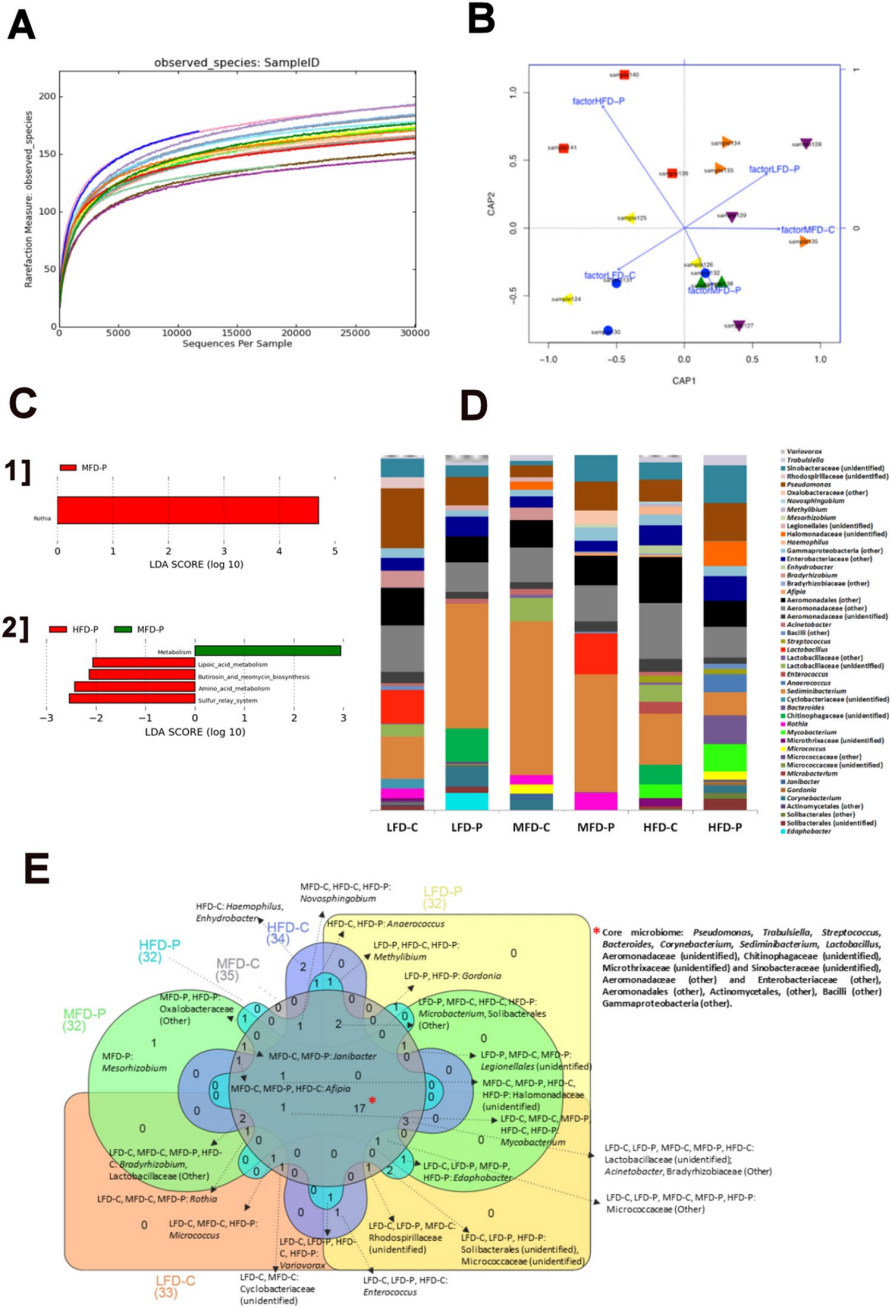
Gastrointestinal bacterial community analysis of zebrafish. (**A**) Alpha rarefaction plot of observed species. (**B**) Distance-based redundancy analysis (db-RDA) using unweighted UniFrac metrics by probiotic supplementation: LFD-P (orange triangules), MFD-C (purple triangules), MFD-P (green triangules), HFD-C (blue circles). (**C**) LEfSe (LDA Effective Size) results of OTUs at genus level (I) and after PiCRUST (phylogenetic investigation of communities by reconstruction of unobserved states) analysis (I) (*p* < 0.05) (**D**) Stacked bar chart representing the relative abundance of bacterial genera. (**E**) Core microbiome at genus level.

High-throughput 16S rRNA V1–2 sequence libraries (performed on 3 samples, each samples made of a pool of 3 fish) generated from the zebrafish gut yielded 681,924 sequence reads (with average length of 254 ± 76 bp), representing 406 OTUs from 1.43 million raw sequence reads. Using USEARCH default parameters, a *de novo* UCHIME algorithm was used to identify potential chimeric sequences, which accounted for 2.9% of the total sequences. Good’s coverage estimators and observed species reached a *plateau* at approximately 5,000 sequences corresponding to 140–190 OTUs (Fig. 7A, Table 1). Shannon diversity indices revealed that community diversity was significantly reduced in the HFD groups compared to both LFD and MFD (Table 1). The spatial study of the community showed a significant separation when the supplementation of the probiotic in the diet was considered in ADONIS, RDA (Fig. 7B), delta and MORANS of the unweighted unifrac distances. When the lipid effect is considered, a significant effect was revealed by PERMANOVA for weighted unifrac distances and in the ADONIS, RDA and delta of the unweighted unifrac distances.

**Table 1:** Alpha diversity metrics of Good’s coverage, Shannon’s, Chao1diversity, phylogenetic diversity and observed species of zebrafish (mean ± s.d.; n = 3, except for LDF-P n = 2).

Diets	Good’s coverage	Shannon	Chao1	Phylogenetic tree	Observed species
LFD-C	0.9979 ± 0.0002	4.73 ± 0.23^abc^	175.32 ± 13.42	4.30 ± 0.31	153.77 ± 11.32
LFD-P	0.9976 ± 0.0001	4.13 ± 0.13^a^	167.95 ± 11.32	4.35 ± 0.38	143.89 ± 11.62
MFD-C	0.9977 ± 0.0002	4.16 ± 0.27^abc^	164.41 ± 13.88	4.08 ± 0.03	141.22 ± 11.59
MFD-P	0.9975 ± 0.0001	4.42 ± 0.05^ab^	174.81 ± 9.37	4.14 ± 0.11	149.60 ± 7.13
HFD-C	0.9977 ± 0.0000	5.10 ± 0.40^bc^	177.55 ± 9.55	4.30 ± 0.72	155.48 ± 10.47
HFD-P	0.9978 ± 0.0005	4.70 ± 0.04^c^	168.84 ± 18.59	4.33 ± 0.63	147.33 ± 13.37

^a,b^Values in the same column with the different superscripts are significantly different (*p* < 0.05).

The bacterial communities were dominated by two phyla: Proteobacteria (accounting for 53.1% of total reads) and Bacteriodetes (28.0% of total reads), with lesser extent Firmicutes (8.6% of total reads), Actinobacteria (8.1% of total reads) and Acidobacteria (2.2% of total reads) (Fig. 7D Linear discriminant analysis effective size (LEfSe) results of the otus.biom table showed that *Rothia* was enriched in treatment MFD-P (Fig. 7C-1). The core microbiota at genus level compromised 17 genera, corresponding to *Pseudomonas*, *Trabulsiella*, *Streptococcus*, *Bacteroides*, *Corynebacterium*, *Sediminibacterium*, *Lactobacillus*, unidentified genera from the families Aeromonadaceae, Chitinophagaceae Microthrixaceae and Sinobacteraceae, and OTUs that could not be classified further than as Aeromonadaceae and Enterobacteriaceae families, than Aeromonadales and Actinomycetales orders, and than Bacilli and Gammaproteobacteria classes. All treatments, with the exception of LFD-P, contained *Mycobacterium*. The LFD fed fish did not contain an unidentified genus of the family Halomonadaceae and *Janibacter* and *Anaerococcus* were only present in communities derived from fish fed the MFD and HFD, respectively. *Mesorhizobium* was unique to the MFD-P fed fish; Oxalobacteraceae (other) was unique to MFD-P and HFD-P fed fish, and *Gordonia* unique in LFD-P and HFD-P fed fish (Fig. 7E).

PiCRUSt analysis was performed and from 81,470 sequences, representing 30 observations after closed-reference OTU picking, PiCRUSt identified 77,222,234 gene markers which correspond to 328 gene ontologies. LEfSe results revealed significant differences in unclassified metabolism in the MFD-P bacterial communities and for the HFD-P communities in metabolism of butirosin and neomycin biosynthesis (biosynthesis of other secondary metabolites), lipoic acid metabolism (metabolism of cofactors and vitamins) and amino acid metabolism (unclassified metabolism), and sulfur relay system (folding, forting and degradation) (*p* < 0.05) (Fig. 7C-2).

### *L*. *rhamnosus* attenuates weight gain in zebrafish fed a HFD and MFD, but not in LFD

We further investigated morphometric analysis on 9 samples per group, each sample made of a pool of 10 adult zebrafish. The treatment with *L*. *rhamnosus* reduced the rate of weight gain in HFD and MFD fed fish. Particularly, results showed that the probiotic reduced the weight gain in HFD-P (47.1 ± 12.2 mg) and MFD-P (46.0 ± 5.5 mg) fed fish compared to HFD-C (73.6 ± 10.2 mg) and MFD-C (64.2 ± 12.7 mg) fed fish (*p* < 0.05), while no significant differences in wet weight were observed between LFD-P (43.5 ± 5.5 mg) and LFD-C (57.1 ± 12.3 mg) fed fish (*p* > 0.05) (Supplemental Table 2).

### PCA analysis

The PCA was performed a on a raw dataset composed of *glp-1*, *npy*, *nucb2*, *hnf4α*, *npc1l1*, *mgll* and *fit2* gene expression and the content of TAG and cholesterol, with the aim of evidencing the correlations among the observed variables.

**Figure 8:**
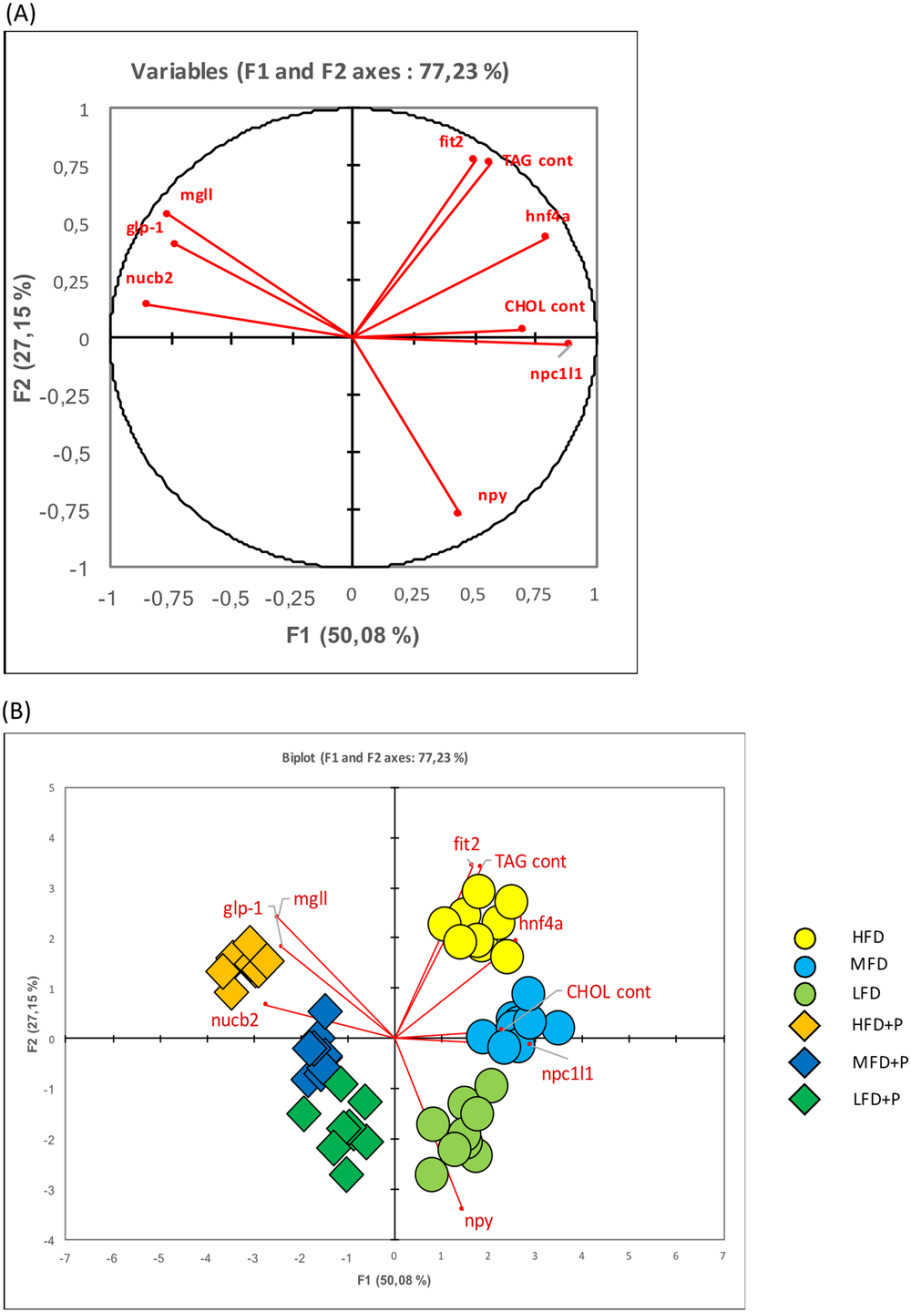
PCA analysis. (**A**) Plot showing how the selected variable correlate with F1 and F2 axes, which were found by the PCA as the two components which best discriminates among groups. (**B**) Plot showing the added observations, which are divided by colours and shapes.

We first report the plot showing how the selected variables correlate with F1 and F2 axes (first and second principal components, respectively), which were found by PCA as the two components which best discriminates among groups (77.23% of cumulative variability explained) (Fig. 8A).

Next, we report the plot of the F1 versus F2 for genes expression analysis and content of TAG and cholesterol. F1 axis discriminates diets with and without probiotic, while F2 axis distinguishes diets based on their lipid content (Fig. 8B).

## Discussion

Due to the therapeutic potential of probiotics for treating dyslipidemia, the mechanisms involved in claming the beneficial effects of probiotics against metabolic disorders have attracted much attention in recent years^1, 28, 29^. Several recent studies have demonstrated that dietary factors (*e*.*g*. elevated dietary content of fats or carbohydrates) shift the microbial community composition which results in biological changes to the host^30, 31^.

In the present study, high throughput-sequencing analysis evidenced specific microbiome core species present in intestines of adult fish fed with different fat contents. We observed that the different lipid content in the diet shifted the gut microbiome structure by reducing community diversity in the HFD group, as well as the variations in the presence or absence of a long list of genera in the gut of fish fed different diets. These modulations, in turn, profoundly modifies triglycerides metabolism and not markedly changed signal involved in appetite control, while no changes were found in respect to total body cholesterol levels. Some evidences both in animal and in human showed that several probiotics strains have hypolipidemic property; however, it is unclear whether probiotics influence the metabolism of the host fed with different fat content. In addition to the effects of fat diets, we showed the capability of probiotic to attenuate weight gain and to decrease cholesterol and TAG content in HFD-P and MFD-P fed fish.

The current results highlight that the different fat content moderately affects the transcription of genes involved in appetite control, while the addition of probiotic decreased the orexigenic signals and increased the anorexigenic signals, thus reducing the response to fasting.

Different studies have showed the emerging role of nesfatin-1 as anorexigenic hormone which has a significant role in appetite control since is able to inhibits NPY, that stimulate food intake^17, 32^. In our study, the expression of anorexigenic *nucb2a*, was lower in MFD-C and significantly upregulated by probiotic, concomitantly with a significant decrease of the orexigen *npy* gene expression in fed a HFD-P and MFD-P groups. In particular, the probiotic decreased NUCB2, the precursor of weight loss inducing peptide nesfatin-1 and enhanced preproghrelin, from which the orexigen is formed. A decrease in NUCB2 suggests its accelerated conversion to processed nesfatin-1, the processed peptide with anorectic and weight loss properties. Similarly, in tissue cross sections, we found less nesfatin-1/NUCB2-like staining in the HFD-P. This is likely due to the fact that less precursor is present (due to its accelerated processing), and more secretion of processed nesfatin-1 from these cells. Meanwhile, an increased accumulation of preproghrelin is reflective of attenuated processing to mature ghrelin that causes hyperphagia and creates a positive energy balance. The net effect of these changes at the transcriptional and post-translational processing of anorexigens and orexigens likely contribute to the weight loss found in adult zebrafish fed probiotics. Due to the small size of adult zebrafish, we used whole head and/or trunk as the sources of RNA and protein used in our analyses. The heterogeneity of tissues sources in the body sections used might have influenced the mRNA and protein expression data, and is a limitation of such studies. The levels of nesfatin-1 and ghrelin were undetectable in our Western blot analyses, while their precursors were detected. In addition, due to small volume of plasma in zebrafish we were not able to measure mature circulating ghrelin which is biologically active^33^.

Furthermore, in the control of appetite, fats triggers the release of several gastrointestinal hormones including glucagon-like peptide 1 (GLP-1) which has anorectic effects^34, 35^. Our findings raise the possibility that the reduction of gene transcripts involved in the control of the appetite could be related to both the capability of the probiotic to act at transcriptional level and to modulate the gut microbiota in a dietary lipid content dependent manner. However, the modulation of the genes that control appetite is not solely attributable to the presumable enhancement of fatty acids produced by microbiota (*e*.*g*. *Lactobacillus* spp. and other lactic acid bacteria), but could be also due to the probiotic’s capability of inducing enteroendocrine L-cell proliferation, thus increment and decrement gut metabolic peptide production and secretion (*e*.*g*. GLP-1, PYY, Ghrelin)^36–38^.

These results are consistent with a previous studies that demonstrated the anorectic effects of probiotics^9, 39^.

In the present study, as previously mentioned, the different lipid content in the diets did not lead to a variation of total body cholesterol; however, the presence of the probiotic in the diets brought about a decrease of the transcription of genes involved in cholesterol metabolism (*hnf4a* and *npc1l1*) and consequently, lowered cholesterol content, in HFD-P and MFD-P fed fish, revealing a hypolipidemic activity of the probiotic. Recent studies evidenced that HNF4a, which is a cholesterol dependent signal, plays a crucial role in the regulation of NPC1L1, a transmembrane protein involved in intestinal cholesterol absorption^20, 21, 40^. Studies on NPC1L1 knockout mice fed a high cholesterol diet demonstrate a reduction in intestinal cholesterol absorption^41, 42^. Due to this, the results obtained here might suggest that the probiotic, by acting at transcriptional level, decreases *hnf4a* mRNA levels which in turn down-regulate *npc1l1*. The down-regulation of the *npc1l1* could lead to a decrease of cholesterol absorption, causing a loss of cholesterol content, showing the probiotic’s capability of preventing fat accumulation induced by lipid content in the diets. In addition, the different lipid content in the diets showed differences in the transcription of genes involved in the synthesis of TAG. We reported that the fish fed a highest fat content diet showed the highest level of transcripts of *mgll* and *fit2*, both involved in the hydrolysis of TAG. Concomitantly we reported that TAG content decreases with the reduction of lipid in the diets. According to Kobyliak *et al*.^43^, we observed that the supplementation of the probiotic in the diets lead to a decrease of the transcription of genes involved in TAG metabolism and, concurrently, our results demonstrated lower TAG content in HFD-P and MFD-P fed fish, but not in LFD-P fed fish.

Recently, monoglyceride lipase (MGLL) has been characterized as the main lipase involved in the final step of TAG hydrolysis, and its knockout showed a reduction in monoacylglycerols hydrolase activity in adipose tissue, brain, and liver^44, 45^. Meanwhile, recent studies on mouse liver and muscle, evidenced that knockdown of *fit2* significantly decreases lipid droplet accumulation, thus reducing fat storage^46^. Remarkably and according to Choi *et al*.^47^ in the present study, the lower TAG content found in zebrafish fed a HFD-P and MFD-P suggests the probiotic’s capability to modulate transcriptional process involved in the regulation of TAG metabolism. Contrary to what previously found in zebrafish larvae^10, 48^, the findings we obtained with adult zebrafish are supported by the loss of weight observed in the adult fish fed a HDF-P and MFD-P compared to the control ones and suggest the capability of the probiotic to successfully prevent weight gain, thus reducing the high-fat-diet induced obesity.

Increasing evidence indicates that a chronic supplementation of high fat content in the diet is associated with an attenuation of the intestinal feedback signals induced by fat, with a consequent modulation of energy intake, lipid accumulation and inflammation^49^.

Furthermore, as seen in the graph (Fig. 8B), F1 axis discriminates the diets dividing them into two groups: those with the probiotic and those without. The variables which most contribute to this division are cholesterol content, *npc1I1*, *nucb2* expression as seen by the centroid plot. F2 axis mostly discriminates diets based on their lipid content. In this case, the variables playing a major role in this separation are TAG content, *fit2* and *npy* expression.

These results show that metabolic changes are mostly related to presence/absence of the probiotic, as this difference is based on the F1 axis (50.08% of variability explained). On the contrary, F2 axis separates metabolic changes according to lipid content in the diet with an explained variability of 27.5%. These results evidenced that the major metabolic changes are induced by probiotic administration rather than lipid content in the diet.

In our study, TEM analysis showed that different content of fat in the diet does not impact the intestine architecture; however, images reveal a consistent accumulation of lipid droplets and infiltration of macrophages in the intestine of fish fed a HFD-C and MFD-C. The addition of the probiotic to the diets did not affect the morphology of the intestinal epithelium and no signs of damage were observed. In fact, the intestine of zebrafish fed HFD-P, MFD-P and LFD-P diets had a similar appearance to the intact intestinal mucosal epithelium, with intracellular tight junctions maintaining epithelial integrity and well organized microvilli, observed in the respective control groups. Despite the lipid droplets accumulation in the intestine of fish fed a elevate and medium fat content, the similar architecture found in the probiotic treated fish could be due to the therapeutic effects of the probiotic that, by modulating intestinal microflora gut, preserve the intestine structure and reduce inflammation as reported by several studies^50–52^.

In conclusion, the results discussed here evidenced that different fat content in the diets leads to a variation in microbiome species, modulate the overall process involved in TAG metabolism, slightly affect appetite control and induce enterocyte lipids droplets formation, but does not interfere with cholesterol metabolism. Moreover, these findings provide information on the nutritional impact of probiotic *L*. *rhamnosus* as a feed additive, indicating some potential to prevent or attenuate obesity and related metabolic disorders. Although application of these findings to clinical populations remains to be validated, the use of this probiotic to beneficially alter the lipid metabolism and food intake appears promising to attenuate high-fat diet-related metabolic disorder.

## Methods

### Ethics Statement

All researches using zebrafish conducted at the University of Marche as well as University of Saskatchewan followed the national guidelines set by the Italian and Canadian Council for Animal Care, and animal research protocols were approved by the institutional Animal Research Ethics Board of University of Marche and by the Ministry of Health of Italy (Protocol #63/INT/CESA12-16). All efforts were made to minimize suffering and a humane endpoint was applied with an excess of anaesthetic (MS222, Sigma- Aldrich, Milano, Italy) when animals reached a moribund state.

### Animals and probiotic administration

Juvenile female and male wild type zebrafish (*Danio* *rerio*) were purchased from Acquario di Bologna (Italy) or from Aquatic Imports (Calgary, Alberta, Canada) and acclimated to the laboratory conditions (27.0 ± 0.5 °C under a 12:12 h light:dark photoperiod). The experiment was set up in triplicates, with three control tanks and three tanks for each diet probiotic enriched and from each tank a pool of adult (three-months old) was collected. The experiment was repeated three times. First and second replicates of the experiment were conducted in Italy, while the third experiment replicate was conducted in Canada.

Two-months old juvenile female and male were divided in tanks and each tank was fed with the different diets. Fish rearing densities were equivalent across all tanks. Fish received fed as 2% of body mass. Six iso-nitrogenous diets were formulated to meet the known nutritional requirements for cyprinids (NRC 2011) with three lipids levels: 5, 10 and 15%, and the enrichment in the probiotic diets of *L*. *rhamnosus* IMC 501® (C025396A; Synbiotec, Camerino, Italy) at a concentration of 10^6^ colony-forming units (CFU). The dry macro ingredients were well mixed. Dietary formulations are presented in Supplementary Table 3. Once homogenised, the micro ingredients (including lyophilized *L*. *rhamnosus* in the probiotic diets), oil and warm water were gradually added to the mixture, prior to pelleting in a PTM P6 extruder (Plymouth, UK). The diets were dried to ca. 5% moisture and stored at 4 °C in airtight containers. Proximate composition was analysed using AOAC (1995) protocols. The experiment was set up in triplicates, with control tanks receiving HFD-C (15% of fat), MFD-C (10% of fat), LFD-C (5% of fat) diets, respectively, and treated tanks receiving HFD-P (15% of fat + probiotic), MFD-P (10% of fat + probiotic), LFD-P diets (5% of fat + probiotic). Fish were fed twice a day for one month.

After one month, zebrafish were anesthetized using MS222 (100 mg L^−1^) (Sigma-Aldrich) and sampling were performed in the morning before giving fed (fasting state). The number of adult utilized is different and depending on the analysis performed. Samples were pooled in order to reach the minimum quantity of the tissue for performing each specific analysis (please see below, each analysis section report further details). Due to the small size of three-months zebrafish, samples were then collected by dividing zebrafish into head and trunk sections for RNA and protein extraction. In addition, samples were collected as the whole fish for high-throughput sequence analysis and High-Performance Liquid Chromatography (HPLC), and intestine samples were sampled for Transmission Electron Microscopy (TEM) and immunohistochemistry (IHC). Samples were stored at −80 °C for Real time PCR analyses, high-throughput sequence analysis and HPLC, while intestine samples were fixed for TEM and IHC. Other samples were stored at −40 °C for Western Blot Analysis (WB).

### DNA extraction, PCR and high throughput-sequencing analysis

DNA was extracted from 3 samples, each sample made of a pool of 3 whole fish (129 ± 18 mg), after surface sterilisation, with a QIAGEN kit following manufacturer’s instructions according to Falcinelli *et al*.^47^. A nested PCR of the 16 S rRNA V1-2 regions was performed on following an external PCR with the primers Eub8F (5′-AGA GTT TGA TCM TGG CTC AG-3′) and 984yR (5′-GTA AGG TTC YTC GCG T-3′); the internal PCR was conducted using the primers 338 R (5′-GCW GCC WCC CGT AGG WGT-3′) and 27 F (5′-AGA GTT TGA TCM TGG CTC AG-3′). Both reactions were carried out in a final volume of 50 μL with 1 μL of DNA template, 25 pmol μL^−1^ of each primer, 25 μL MyTaq^TM^ (Bioline, London, UK) and 22 μL of molecular grade water in a TC-512 thermal cycler (Techne, Staffordshire, UK). For the external PCR: 25 cycles of 95 °C for 30 s, 50 °C for 30 s, 72 °C for 1 min and a final extension 72 °C for 5 min. The annealing temperature was 50 °C. For the internal PCR: initial denaturation at 94 °C for 7 min, then 10 cycles at 94 °C for 30 s, followed by a touchdown of 1 °C per cycle from 62 −53 °C for 30 s and 72 °C for 30 s. A further 20 cycles were performed at 94 °C for 30 s, 53 °C for 30 s, 72 °C for 30 s and a final extension 72 °C for 7 min.

Amplicons were purified with Ampure beads (0.8×) and were sequenced using a 318^TM^ chip (LifeTechnologies^TM^) on an Ion Torrent Personal Genome Machine (LifeTechnologies^TM^) at the Systems Biology Centre in Plymouth University (UK) as described by Falcineli *et al*., 2016. High-throughput sequencing fastQ files were analysed, after the removal of low quality scores (Q score < 20 at 80% probability) with FASTX-Toolkit (Hannon Lab, USA), using QIIME 1.8 with default parameters at 97% similarity against 13_8 Greengenes database. Relative abundance less than 0.1% was not included in the analysis according to Jervis-Bardy *et al*. (2015). The Class Chloroplast (48 sequences) and Genus *Propionibacterium* (12,909 sequences) were considered contaminants^51^ and were removed from the otus.biom table before alpha and beta analysis. PICRUSt analysis was performed using Greengenes database gg_13_5 at 97% similarity as closed-reference OTU picking, all unassigned sequences were discarded.

Alpha and beta diversity metrics and distances were calculated on rarefied OTU tables with QIIME to assess sampling depth coverage using observed species and Good’s coverage, phylogenetic diversity, Chao1, Shannon’s diversity indexes, weighted and unweighted Unifrac distances were also calculated. The distance matrixes were represented by a Distance-based redundancy analysis (db-RDA) plot.

### RNA extraction and cDNA synthesis

From 9 samples per group (each sample made of a pool of 8 adult’s head or trunk, depending on the site of the gene expression), total RNA was extracted from zebrafish head and trunk using an RNAeasy® minikit (QIAGEN, UK) following the manufacturer’s protocol. The extracted RNA from was eluted in 15 µL of RNAse-free water. Final RNA concentrations were determined using a Nanophotometer TMP-Class (Implem GmbH, Munich, Germany) and the RNA integrity was verified by ethidium bromide staining of 28S and 18S ribosomal RNA bands on a 1% agarose gel. RNA was stored at −80 °C until use. A total amount of 1 µg of RNA was used for cDNA synthesis, employing an iScript cDNA Synthesis Kit (Bio-Rad laboratories, USA).

### Real time PCR

PCRs were performed with the SYBR green method in an iQ5 iCycler thermal cycler (Bio-Rad laboratories). Triplicate PCRs were carried out for each sample following exactly Maradonna *et al*.^53^. In addition, in order to standardize the results by eliminating variation in mRNA and cDNA quantity and quality^54^, *β-actin* (*actβ*)^55^ and *acidic ribosomal protein* (*arp*)^56^ were used as the reference genes. The choice of reference genes were due based of the fact that their mRNA levels did not vary among developmental stages and experimental treatments. Results demonstrated neither amplification product produced in negative controls and nor primer-dimer formations in the control templates. The data obtained were analyzed using the iQ5 optical system software version 2.0 (Bio-Rad laboratories). Modification of gene expression is reported with respect to the control sample. The primer sequences for *actβ*, *rplp*, *mgll*, *hnf4a*, *fit2*, *npy*, *gcga* and *nucb2a* were designed using Primer3 (210 v. 0.4.0) (Supplemental Table 4).

### High-performance liquid chromatography (HPLC)

The total lipid contents were extracted from 9 samples, each sample made of a pool of 300 mg of adult zebrafish and cholesterol and TAG were analysed according to Falcinelli *et al*.^10^ with a HPLC-Light Scattering Detector- LC-10AD VP (Shimadzu, Kyoto, Japan).

### Western blot analysis

Four samples, each sample made of a adult zebrafish trunk were homogenized in T-PER® tissue protein extraction reagent (Thermo Scientific, #78510) followed by measurement of protein concentration by Bradford assay. The samples were prepared in 1X Laemmli buffer containing 5% 2-mercaptoethanol (Bio-Rad, #161-0737 and -0710) and subsequently were boiled at 95 °C for 5 min followed by vortexing. The whole sample volume (20 µL) each containing 50 µg protein was loaded and run in a Mini-PROTEAN® TGX™ 4–20% gradient gel (Bio-Rad, #456-1096) at 200 V for 30 min. After separation the proteins were transferred to a 0.2 µm BioTrace™ nitrocellulose membrane (PALL Life Sciences, #27377-000) at 100 V for 40 min. membrane was blocked in 1X RapidBlock™ solution (aMReSCO, #M325). NUCB2 protein detection was performed using rabbit antiserum directed against mouse NUCB2 (1312-PAC-01 custom antibody, Pacific Immunology) diluted 1:1000. Vinculin protein was detected by use of rabbit antiserum directed against mouse Vinculin (Abcam, # Ab73412) diluted 1:3000 and ghrelin was detected by use of goat antiserum directed against mouse gherlin (Abcam, # 57222) overnight at 4 C. After washing membrane exposed to secondary antibody goat anti-rabbit IgG (H + L) HRP conjugate (Bio-Rad, #170-6515) diluted 1:3000 was used for NUCB2 and Vinculin and goat anti-mouse IgG (H + L) HRP conjugate (Bio-Rad, #171-1011)was used for ghrelin. For protein visualization the membrane was incubated for 5 min in Clarity™ western ECL substrate (Bio-Rad, #170-5061) and imaged using ChemiDoc™ MP imaging system (Bio-Rad, #170-8280) with chemiluminescence detection. Membrane stripping in between protein detection was conducted using Restore™ PLUS western blot stripping buffer (Thermo Scientific, #46430). Precision plus protein™ dual xtra standards (Bio-Rad, #161-0377) were used as molecular weight markers.

### NUCB2/Nesfatin-1 and ghrelin- like immunoreactivity in zebrafish intestine

Immunofluorescence study was performed on 4 fish exactly as described previously^11, 57^. Briefly, paraffin-embedded sections (10 sections per fish) were deparaffinized in 100% xylene for 10 min and re-hydrated in graded ethanol series. Phosphate buffered saline (PBS) and antibody-blocking solution (ABB) was used for blocking for 10 min. The sections were then incubated in rabbit anti-nesfatin-1 primary polyclonal antibody (Catalog # H-003- 22, Pheonix Pharmaceuticals, California, 1:1000 dilution) and mouse anti- ghrelin primary monoclonal antibody (Catalog # ab57222, Abcam, Massachusetts; 1:500 dilution) overnight at 4 C. After incubation the slides were washed thrice in 1x PBS for 10 min and incubated with goat anti-rabbit IgG (Texas Red, Nesfatin-1; Catalog # TI-1000, Vector Laboratories, CA; 1:500 dilution) and goat anti- mouse. IgG (FITC, ghrelin, Catalog # ab6785, Abcam, 1:500 dilution) secondary antibody for 1 h at room temperature. Slides were washed thrice in 1x PBS for 10 min and dried in a dark chamber for 10 min at room temperature. Slides were mounted using Vectashield mounting medium containing the DAPI nuclear fluorescent dye. The negative controls were not incubated with primary antibodies and were only treated with secondary antibody. The images were taken using Nikon inverted microscope (L100) (Nikon, Canada) and NiS Elements microscope imaging software (Nikon, Canada). Only representative images for nesfatin-1 and ghrelin are shown. The total number of all intestinal epithelial cells immunoreactive for nesfatin-1 alone (red) or ghrelin alone (green) was counted in all sections assessed. To calculate the percentage of distribution, the total number of positive cells under each category (ghrelin/nesfatin-1 positive) was divided by the total number of cells and result was multiplied by 100 to obtain the percentage population of positive cells.

### Transmission Electron Microscopy (TEM)

Samples of 9 adult zebrafish intestines, for each experimental group were fixed, prepared and observed by TEM (JEOL 1200 EXII, JEOL Tokyo, Japan) according to Falcinelli *et al*.^10^.

### Morphometrical analysis

Wet weight was determined from 9 samples, each sample made of a pool of 10 adult zebrafish, using a microbalance (OHAUS Explorer E11140, Pine Brook, NJ, USA).

### Statistical analysis

Results were expressed as the mean ± s.d. Statistical differences were determined using ANOVA, followed by Bonferroni’s multiple comparison test. All statistical analyses were performed using Prism 6 (GraphPad Software, San Diego, CA, USA). Predictive functional composition analysis of the bacterial communities was conducted on 16S rRNA microbiomic data using PiCRUSt according to Langille *et al*.^58^. Linear discriminant analysis effective size (LEfSe) was used to identify significant differences with biological consistency and effect size estimation in relative abundance of bacterial taxa and KEGG orthologs according to Segata *et al*. (2010). Vegan and ape packages from R were used to analyse beta diversity data. P-values < 0.05 were considered significant.

PCA was performed by using XLSTAT program based the Pearson’s correlation matrix in order to summarize the correlations among the set of observed variables. A raw dataset composed of the observed variables such as gene expression of *glp-1*, *npy*, *nucb2*, *hnf4α*, *npc1l1*, *mgll*, *fit2*, and the content of TAG and CHOL, were took into account.

## Electronic supplementary material

supplemental information
