# Supplementary material for: Dietary lipid content reorganizes gut microbiota and probiotic L. rhamnosus attenuates obesity and enhances catabolic hormonal milieu in zebrafish
Source: Sci Rep. 2017 Jul 17;7:5512. doi: 10.1038/s41598-017-05147-w (PMC5514052; doi:10.1038/s41598-017-05147-w)
Supplement: Supplementary file 1 — supplemental information [file 41598_2017_5147_MOESM1_ESM.pdf]

Dietary lipid content reorganizes gut microbiota and probiotic *L. rhamnosus* attenuates obesity and enhances catabolic hormonal milieu in zebrafish.

Silvia Falcinelli[1], Ana Rodiles[2], Azadeh Hatef[3], Simona Picchietti[4], Lina Cossignani[5], Daniel L. Merrifield[2], Suraj Unniappan[3] and Oliana Carnevali[1*]

Supplemental table S.2.

The probiotic *L. rhamnosus* changes weight in zebrafish fed a HFD and MFD.

Diets	Control	Probiotic
**HFD**	73.6 ± 10.2 mg	47.1 ± 12.2 mg*
**MFD**	64.2 ± 12.7 mg	46.0 ± 5.5 mg*
**LFD**	57.1 ± 12.3 mg	43.5 ± 5.5 mg

Values with asterisk are significantly different ($P < 0.05$).

Supplemental table S.3

Composition Chart of 5% fat diet (LFD)

Ingredient Restrictions

Ingredient	Min(%)	Max(%)	Usage(%)	Batch(kg)
Soyabean (Hamlet HP100)			44.62	446.17
Corn Starch			31.22	312.23
Herring Meal LT94	15.00	15.00	15.00	150.00
Dried Brewers Yeast	5.00	5.00	5.00	50.00
Fish oil			2.56	25.59
Vitamin Premix	1.00	1.00	1.00	10.00

CMC-binder	0.50	0.50	0.50	5.00
Ascorbyl-Phosphate (DSM)	0.10	0.10	0.10	1.00

Nutrient Restrictions

Nutrient	Units	Min Limit	Max Limit	Actual	Shadow
01. Dry Matter (DM)	%		100.00	94.93	
02. Crude Protein	%	38.00	38.00	38.00	-0.00
03. DCP	%			9.24	
04. Crude lipid	%	5.00	5.00	5.00	0.01
06. Crude Fiber	%			1.22	0.00
07. Phosphorous	%			0.64	
08. Calcium	%			0.46	
09. Magnesium	%			0.19	
10. Avail. Phos.	%			0.25	
11. Ash	%			3.03	
12. Lysine	%			2.42	
13. Methionine	%			0.63	
14. Met + Cys	%			0.42	
15. Arginine	%			2.56	-0.00
16. Histidine	%			0.92	
17. Threonine	%			1.45	
18. Tryptophan	%			0.47	
19. Leucine	%			2.78	0.00

20. Isoleucine	%			1.67	
21. Phenylalanine	%			1.70	
22. Valine	%			1.89	
24. Vitamin E	mg/kg			44.60	
26. Choline	mg/g			1.54	
27. Vitamin C	mg/kg			0.05	

Composition Chart of 10% fat diet (MFD)

Ingredient Restrictions

Ingredient	Min(%)	Max(%)	Usage(%)	Batch(kg)
Soyabean (Hamlet HP100)			44.62	446.17
Corn Starch			26.22	262.23
Herring Meal LT94	15.00	15.00	15.00	150.00
Fish oil			7.56	75.59
Dried Brewers Yeast	5.00	5.00	5.00	50.00
Vitamin Premix	1.00	1.00	1.00	10.00
CMC-binder	0.50	0.50	0.50	5.00
Ascorbyl-Phosphate (DSM)	0.10	0.10	0.10	1.00

Nutrient Restrictions

Nutrient	Units	Min Limit	Max Limit	Actual	Shadow
01. Dry Matter (DM)	%		100.00	94.93	
02. Crude Protein	%	38.00	38.00	38.00	-0.00

03. DCP	%			9.24	
04. Crude lipid	%	10.00	10.00	10.00	0.01
06. Crude Fiber	%			1.22	0.00
07. Phosphorous	%			0.64	
08. Calcium	%			0.46	
09. Magnesium	%			0.19	
10. Avail. Phos.	%			0.25	
11. Ash	%			3.03	
12. Lysine	%			2.42	
13. Methionine	%			0.63	
14. Met + Cys	%			0.42	
15. Arginine	%			2.56	-0.00
16. Histidine	%			0.92	
17. Threonine	%			1.45	
18. Tryptophan	%			0.47	
19. Leucine	%			2.78	0.00
20. Isoleucine	%			1.67	
21. Phenylalanine	%			1.70	
22. Valine	%			1.89	
24. Vitamin E	mg/kg			44.60	
26. Choline	mg/g			1.54	
27. Vitamin C	mg/kg			0.05	

Composition Chart of 15% fat diet (HFD)

Ingredient Restrictions

Ingredient	Min(%)	Max(%)	Usage(%)	Batch(kg)
Soyabean (Hamlet HP100)			44.62	446.17
Corn Starch			21.22	212.23
Herring Meal LT94	15.00	15.00	15.00	150.00
Fish oil			12.56	125.59
Dried Brewers Yeast	5.00	5.00	5.00	50.00
Vitamin Premix	1.00	1.00	1.00	10.00
CMC-binder	0.50	0.50	0.50	5.00
Ascorbyl-Phosphate (DSM)	0.10	0.10	0.10	1.00

Nutrient Restrictions

Nutrient	Units	Min Limit	Max Limit	Actual	Shadow
01. Dry Matter (DM)	%		100.00	94.93	
02. Crude Protein	%	38.00	38.00	38.00	-0.00
03. DCP	%			9.24	
04. Crude lipid	%	15.00	15.00	15.00	0.01
06. Crude Fiber	%			1.22	0.00
07. Phosphorous	%			0.64	
08. Calcium	%			0.46	
09. Magnesium	%			0.19	
10. Avail. Phos.	%			0.25	

11. Ash	%			3.03	
12. Lysine	%			2.42	
13. Methionine	%			0.63	
14. Met + Cys	%			0.42	
15. Arginine	%			2.56	-0.00
16. Histidine	%			0.92	
17. Threonine	%			1.45	
18. Tryptophan	%			0.47	
19. Leucine	%			2.78	0.00
20. Isoleucine	%			1.67	
21. Phenylalanine	%			1.70	
22. Valine	%			1.89	
24. Vitamin E	mg/kg			44.60	
26. Choline	mg/g			1.54	
27. Vitamin C	mg/kg			0.05	

Supplemental table S.4.

Primer sequences used for real-time polymerase chain reaction analyses

Gene	Forward	Reverse
nucb2a	**5’- AGGAGCGGCATGAAGAATTT -3’**	**5’- GATGGTTGACTTTGGGGTGA -3’**
gcga	**5’- CAGAGGGAACTTTCTCCAACGA -3’**	**5’- AGTTCATACCCATTTCTCTTAGCGT -3’**
npy	**5’- GTCTGCTTGGGGACTCTCAC -3’**	**5’- CGGGACTCTGTTTCACCAAT -3’**
npc1l1	**5’-GTCGGAAGGGTAAGAGCACC -3’**	**5’-ATCACAAGGCAGACCGGAAG -3’**
mgll	**5’- CGAGAGGCCGCAGGATTTTA -3’**	**5’- TGAGTTTAGGAGCCAAGCG -3’**
fit2	**5’- CTGGTCTCCCTCCACAGCCGA -3’**	**5’- ACACCAGCTGCCCTCCGCTT -3’**
hnf4a	**5’- ACGGTTCGGCGAGCTGCTTC -3’**	**5’- TCCTGGACCAGATGGGGGTGT -3’**
rplp	**5’-CTGAACATCTCGCCCTTCTC -3’**	**5’-TAGCCGATCTGCAGACACAC-3’**
β-act	**5’ -GGTACCCATCTCCTGCTCCAA-3’**	**5’-GAGCGTGGCTACTCCTTCACC-3’**

SUPPLEMENTAL FIGURE AND FIGURE LEGEND

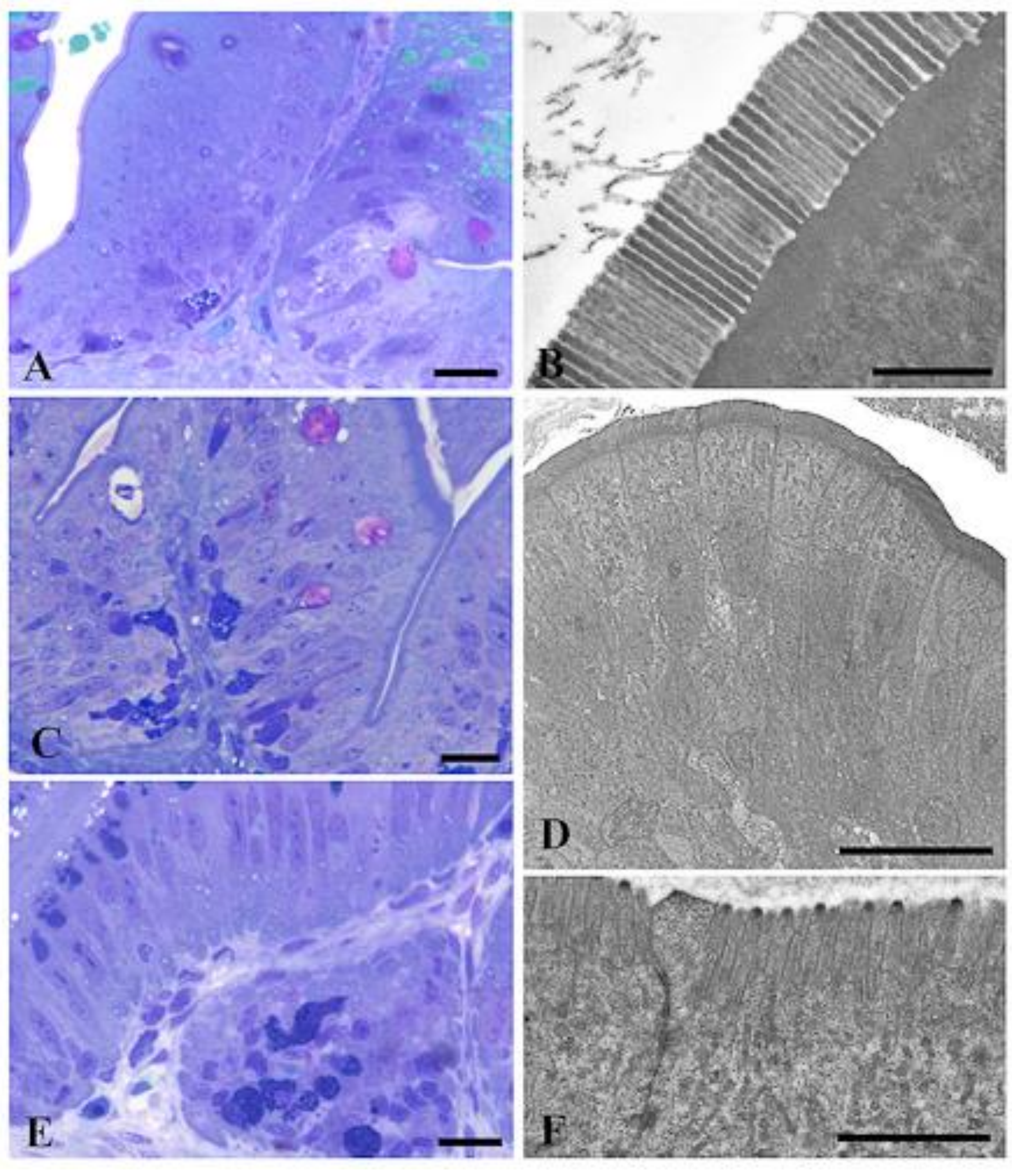

Supplemental Fig 6. ***Lactobacillus rhamnosus*** **did not alter intestinal mucosal epithelium in zebrafish fed a HFD-P, MFD-P and LFD-P.**

Micrographs showed the absence of damage signs in the intestine of fish fed with diet probiotic enriched (Figs. 6 A-F).

Micrograph also showed an intact intestinal mucosal epithelium with intracellular tight junctions epithelial integrity, well organized microvilli and no cell debris in the intestinal lumen of zebrafish fed a HFD-P, MFD-P and LFD-P (Fig. 6 B, D, F).

Scale bar: A, C, D ed E = 10 µm, B ed F = 1 µm
